# Possible Molecular Mechanisms Underlying the Decrease in the Antibacterial Activity of Protamine-like Proteins after Exposure of *Mytilus galloprovincialis* to Chromium and Mercury

**DOI:** 10.3390/ijms24119345

**Published:** 2023-05-26

**Authors:** Carmela Marinaro, Gennaro Lettieri, Mariavittoria Verrillo, Michela Morelli, Federica Carraturo, Marco Guida, Marina Piscopo

**Affiliations:** 1 Department of Biology, University of Naples Federico II, 80126 Napoli, Italymarco.guida@unina.it (M.G.); 2 Department of Agricultural Sciences, Interdepartmental Research Centre of Nuclear Magnetic Resonance for the Environment, AgriFood and New Materials (CERMANU), University of Naples Federico II, 80055 Portici, Italy

**Keywords:** antibacterial activity, sperm proteins, protamine-like proteins, *Mytilus galloprovincialis*, gram positive, gram negative, natural molecules, heavy metals, fluorescence measurement

## Abstract

Natural bioactive compounds represent a new frontier of antimicrobial molecules, and the marine ecosystem represents a new challenge in this regard. In the present work, we evaluated the possibility of changes in the antibacterial activity of protamine-like (PL) proteins, the major nuclear basic protein components of *Mytilus galloprovincialis* sperm chromatin, after the exposure of mussels to subtoxic doses of chromium (VI) (1, 10, and 100 nM) and mercury (1, 10, and 100 pM) HgCl_2_, since these metals affect some properties of PL. After exposure, we analyzed the electrophoretic pattern of PLs by both acetic acid-urea polyacrylamide gel electrophoresis (AU-PAGE) and SDS-PAGE and determined the MIC and MBC of these proteins on different gram+ and gram– bacteria. PLs, particularly after mussels were exposed to the highest doses of chromium and mercury, showed significantly reduced antibacterial activity. Just at the highest doses of exposure to the two metals, changes were found in the electrophoretic pattern of PLs, suggesting that there were conformational changes in these proteins, which were confirmed by the fluorescence measurements of PLs. These results provide the first evidence of a reduction in the antibacterial activity of these proteins following the exposure of mussels to these metals. Based on the results, hypothetical molecular mechanisms that could explain the decrease in the antibacterial activity of PLs are discussed.

## 1. Introduction

Antimicrobial resistance is becoming increasingly serious with the abuse of antibiotics in medicine, agriculture, and animal husbandry, especially in developing countries [1]. It has been estimated that the number of deaths attributed to antibiotic resistance will reach 10 million per annum by 2050 if no action is taken [2]. In this context, an inappropriate use of antibiotics, in both human and veterinary medicine, as well as in some food production systems, performed a selective pressure on bacterial strains, which encode several mechanisms to survive in the presence of antibiotics [3]. In recent years, considerable effort has been dedicated to developing compounds that are both highly efficient and also less susceptible to bacterial resistance development. In the past two decades, attention has been paid to natural products as possible sources of new bactericidal agents. Antimicrobial peptides (AMPs) are a class of small peptides that widely exist in nature, and they are an important part of the innate immune system of different organisms [4]. Peptides and proteins have a wide range of inhibitory effects against bacteria, fungi, parasites, and viruses and can be extracted from different sources, such as plants, vertebrates, and invertebrates [5]. Marine organisms represent a valuable source of new compounds. The biodiversity of the marine environment and the associated chemical diversity constitute a practically unlimited resource of new active substances in the field of development of bioactive products [6,7]. Furthermore, very different kinds of substances have been obtained from marine organisms, mainly because they live in very exigent, competitive, and aggressive surroundings, completely different, in many aspects, from the terrestrial environment, a situation that demands the production of specific and potent active molecules [8,9]. *M. galloprovincialis* is one of the most suitable organisms for biomonitoring programs; it is also employed as a bioindicator to track the reproductive health of organisms [10,11,12,13]. The sperm chromatin of this organism is mainly organized by three protamine-like proteins (PLs), PL-II, PL-III, and PL-IV, and we have already demonstrated, for the first time, the antibacterial activity of these proteins against various Gram-positive and Gram-negative bacteria [14]. Despite the important role played by PLs, these proteins are extremely sensitive to the exposure of *M. galloprovincialis* to various pollutants, including heavy metals [15,16]. In fact, we have shown that exposure of this mussel to some metals [17,18,19], but in particular chromium and mercury, can alter some properties of PLs [15,16,19,20]. As is well known, the heavy metals have proven to be a major threat to organism health, mostly because of their ability to perturb protein function and enzyme activity [21].

Furthermore, it has been shown that in the Mediterranean mussel *Donax trunculus*, the activity of enzymes, such as dehydrogenases (lactate and malate dehydrogenase), respiratory enzymes (cytochrome oxidase), and digestive enzymes (α-amylase) is reduced after exposure of mussels to certain heavy metals [22]. For these reasons, and also because there are currently no data in the literature on possible changes in the antibacterial activity of these proteins after exposure of *M. galloprovincialis* to heavy metals, in the present work, we aimed to verify the maintenance of the antibacterial activity of the crude extract of PL containing PL-II, PL-III, and PL-IV after the exposure of mussels to different doses of HgCl_2_ and Cr(VI).

## 2. Results

### 2.1. Electrophoretic Analysis of the PLs of M. galloprovincialis

**Figure 1:**
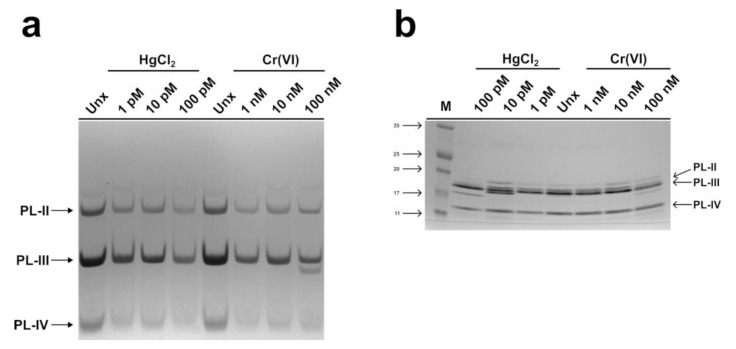
Electrophoretic pattern analysis of PLs by AU-PAGE (**a**) and SDS-PAGE (**b**). PLs extracted from unexposed and exposed mussels to 1, 10, and 100 pM HgCl_2_; 1, 10, and 100 nM Cr(VI). Unx = unexposed condition (control condition); M = molecular weight marker.

The electrophoretic pattern of PLs from spermatozoa of *M. galloprovincialis* exposed to HgCl_2_ (1, 10, and 100 pM) and Cr(VI) (1, 10, and 100 nM) was evaluated on acid acetic-urea polyacrylamide gel (AU-PAGE) and sodium dodecyl sulphate-polyacrylamide gel (SDS-PAGE). The AU-PAGE did not show significant differences in the PLs patterns caused by the two heavy metals with respect to the PLs from unexposed mussels (Figure 1a). The electrophoretic pattern of PLs in SDS-PAGE, instead, showed some differences, particularly for PL, after the exposure of mussels to HgCl_2_ and the co-migration of PL-II with PL-III at the highest exposure dose for both metals; however, the difference was always more pronounced in the case of HgCl_2_. In fact, the intensity of the band migrating at the level of PL-III was more intense than in the no-exposure condition, and PL-II was not visible anymore (Figure 1b).

### 2.2. Determination of MIC and MBC of the PLs of M. galloprovincialis

**Figure 2:**
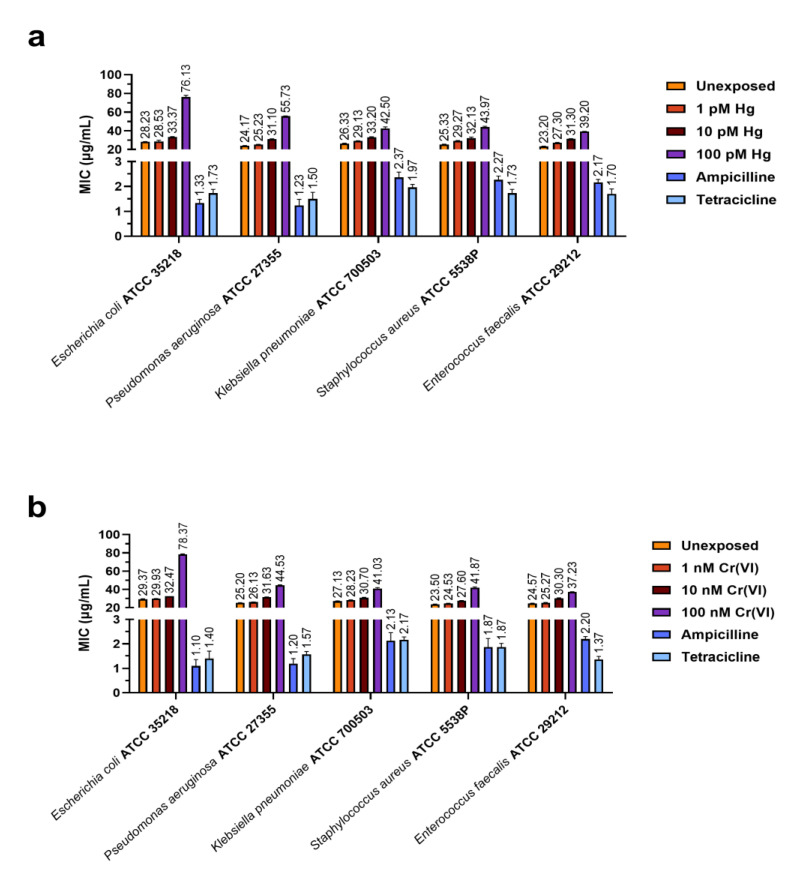
Histograms show the MIC value (µg/mL) of PLs from the mussels exposed to HgCl_2_ (1, 10, and 100 pM) (**a**) and Cr(VI) (1, 10, and 100 nM) (**b**). The graph also shows MIC values for two antibiotics as positive controls: ampicillin and tetracycline. The values reported in the graph are a mean ± S.D. of 3 measurements.

**Figure 3:**
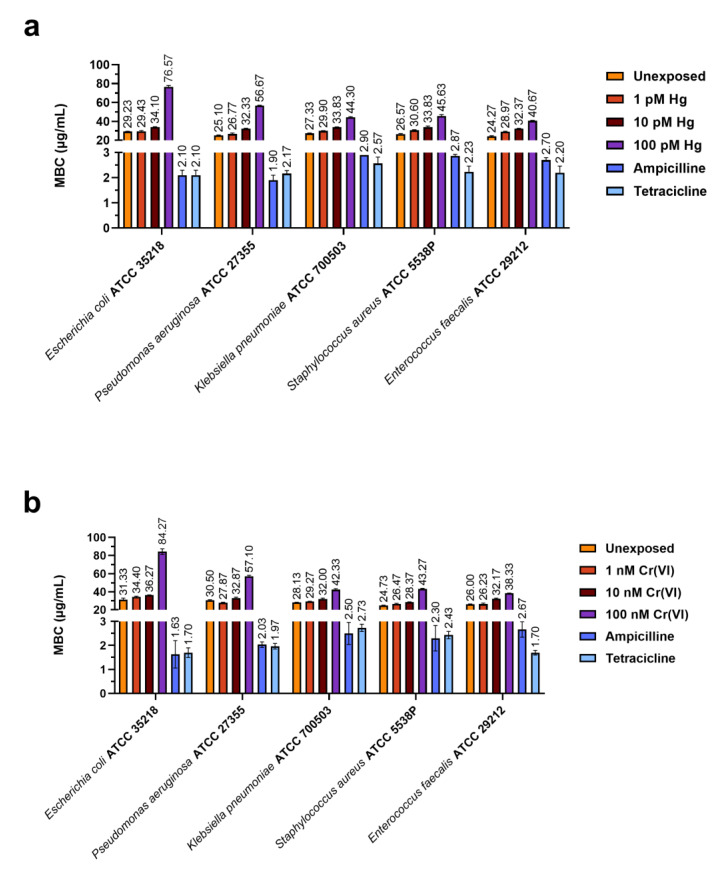
Histograms show the MBC value (µg/mL) of PLs from the mussels exposed to HgCl_2_ (1, 10, and 100 pM) (**a**) and Cr(VI) (1, 10, and 100 nM) (**b**). The graph also shows MBC values for two antibiotics as positive controls: ampicillin and tetracycline. The values reported in the graph are a mean ± S.D. of 3 measurements.

Figure 2 and Figure 3 show the values of minimum inhibitory concentration (MIC) and minimum bactericidal concentration (MBC) values, respectively, of PL extracted from mussels exposed to the two heavy metals that were tested. Panels a and b in each figure demonstrate the results obtained for PLs of mussels exposed to HgCl_2_ and Cr(VI), respectively.

**Figure 4:**
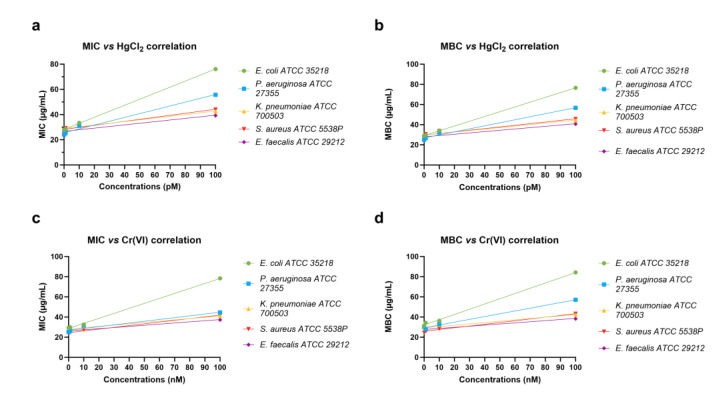
Pearson’s correlation between heavy metal doses and MIC and MBC values obtained by PL for each bacterial strain. (**a**) MIC HgCl_2_ vs. 1, 10, and 100 pM HgCl_2_; (**b**) MBC HgCl_2_ vs. 1, 10, and 100 pM HgCl_2_; (**c**) MIC Cr(VI) vs. 1, 10, and 100 nM Cr(VI); (**d**) MBC Cr(VI) vs. 1, 10, and 100 nM Cr(VI).

For both heavy metals, the MIC and MBC values for the lowest (1 pM HgCl_2_ and 1 nM Cr(VI)) and intermediate (10 pM HgCl_2_ and 10 nM Cr(VI)) concentrations were similar to the MIC and MBC values obtained with the PLs of unexposed mussels (control condition), respectively, for each bacterial strain. For the highest concentrations (100 pM HgCl_2_ and 100 nM Cr(VI)), instead, we observed an increase in the MIC and MBC values, particularly for *E. coli* ATCC 35218. Figure 4 shows the Pearson correlations between the heavy metals tested, MBC and MIC values, obtained with PLs from mussels exposed and unexposed to heavy metals. In addition, Table 1, Table 2, Table 3 and Table 4 show the statistical analyses of the Pearson correlation. Notably, for *E. coli* ATCC 35218, the analysis showed a significant correlation between the highest concentrations of HgCl_2_ and Cr(VI), 100 pM and 100 nM, respectively, and the MIC and MBC values. The statistical analyses of the MIC and MBC values, produced by a two-way ANOVA, are reported in the Supplementary Materials.

### 2.3. Fluorescence Analyses of PLs

**Figure 5:**
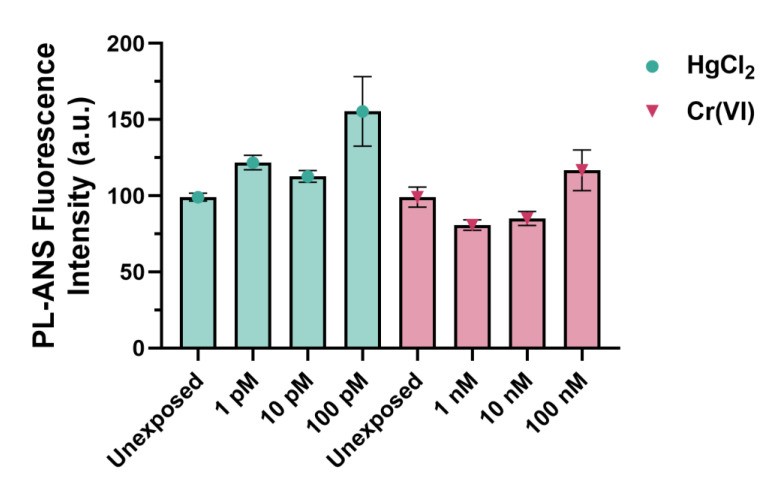
Histograms showing the changes in fluorescence intensity of PLs of mussels exposed to HgCl_2_ (1, 10, and 100 pM) and Cr(VI) (1, 10, and 100 nM) compared to the non-exposure (control) condition. Histograms show the maximum fluorescence for each dose of the two heavy metals used. Values are reported as mean ± S.D. of 3 measurements.

Fluorescence analyses of PLs in the presence of 8-anilino-1-naphthalenesulfonic acid (ANS) have shown differences in the PLs–ANS complex fluorescence intensity for PLs from mussels exposed to each heavy metal tested (Figure 5). An increased fluorescence intensity of the PLs–ANS complex was observed when exposed to HgCl_2_, for all doses but particularly at the 100 pM dose of HgCl_2_, compared to PLs under non-exposure conditions (Figure 5). In contrast, for PLs of mussels exposed to Cr(VI), there was a decrease in the fluorescence of the PLs–ANS complex under conditions of 1 and 10 nM Cr(VI), but an increase at 100 nM Cr(VI) compared to the non-exposure condition (Figure 5). In general, the fluorescence values of the PLs–ANS complex for the HgCl_2_ exposure condition were higher compared to those of the Cr(VI) exposures.

### 2.4. Thermal Stability of PLs

**Figure 6:**
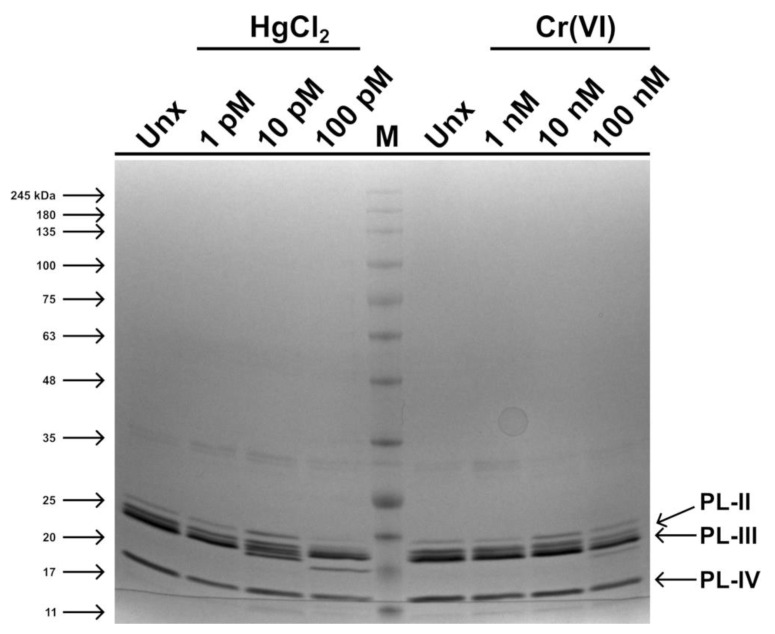
Analysis of the SDS-PAGE 4–20% gradient gel of PLs after 18 h at 37 °C. Unx = unexposed condition (control condition); M = molecular weight marker.

Figure 6 shows the electrophoretic patter of PLs in SDS-PAGE after incubation at 37 °C for 18 h to test the thermal stability of the PLs. The analysis does not show a differences between the different exposure conditions.

## 3. Discussion

For several decades, terrestrial environments were the focus of research concerning the discovery of natural compounds that could be used in the pharmaceutical industry. Indeed, it is well known that the marine environment is a rich storehouse for new bioactive natural products whose structure and chemical properties are not typically found in the natural products of the terrestrial environment [23,24]. Additionally, it is from the marine environment that the molecules that are the subject of this experimentation are derived. The antibacterial activity of *M. galloprovincialis* PLs against various Gram-positive and Gram-negative bacterial strains and also on the clinical isolates of these bacteria was reported for the first time in 2018 [14]. In that work, the antibacterial activity of the crude PLs extract containing all three PLs, i.e., PL-lI, PL-III, PL-IV, and individual PLs, was analyzed, and it was found that these proteins were more active together than individually [14]. Since then, much research has been devoted to the effects of mussel exposure to various heavy metals on the properties of PL. This is because the heavy metal contamination of the marine environment has increased in recent years due to the growth of the world population and industrial development [25]. In addition, metals are of particular concern among environmental contaminants because they have extensive effects on ecosystems, are poisonous, and bioaccumulate in ecosystems, biological tissues, and organs [26]. In our previous studies, we have shown that many heavy metals had marked effects on PLs at the expense of their function, which is rather worrying because of their role in the organization of sperm DNA in *M. galloprovincialis* [13,15,16,17,19,27]. After all, it has been reported in the literature that some heavy metals can inhibit soil enzyme activity and can influence the spreading of AMPs in both negative and positive ways [28]. In fact, pollutant concentrations and soil enzyme activity have generally been shown to have a negative link [29]. The alterations in the properties of PLs that we detected after the exposure of mussels to certain heavy metals prompted us to investigate whether the antibacterial activity of these proteins could also be affected in some way after the exposure of mussels to those metals (chromium and mercury), which had produced greater changes in the other properties of these proteins. In the present work, we conducted the analyses directly on the crude extract containing the three PLs, since in previous work, it had been shown to be more active than the individual PLs [14]. The protamine-like proteins were extracted with 5% PCA because they belonged to the group of H1 histones, and in the same way, H1 histones are soluble in PCA 5%. We found that on various bacterial strains, the maximum exposure dose tested for both mercury and chromium produced a reduction in the antibacterial activity of these proteins. We questioned why such a thing could happen and then analyzed whether PLs had undergone possible conformational changes as a result of the exposure of mussels to these metals. The protamine-like proteins are particularly rich in arginine and lysine residues; therefore, a valuation of their molecular weight by SDS-PAGE is very difficult because these proteins migrate not only on the basis of their molecular weight but also for their charge. Nevertheless, we found alterations in the electrophoretic pattern of these proteins caused by SDS-PAGE, particularly after the exposure of mussels to the highest doses of chromium and mercury. Specifically, we observed a comigration of PL-II with PL-III in SDS-PAGE in samples obtained after the exposure of mussels to the highest doses of both metals but in particular to HgCl_2_. Differences in the intensity of PL-IV have been observed, which could be attributed to the fact that this protein undergoes post-translational modifications, such as ADP-ribosylation, upon the exposure of mussels to these metals, as revealed by our very recent experiments (manuscript in preparation). Since amino acids that are ADP-ribosylated include lysines and arginines, which are known to be amino acids that are bound by Coomassie, and therefore, it is possible that there is interference with the dye. Although a change in the arrangement of bands on electrophoresis cannot be a direct consequence of proteins’ conformation changes, we conducted fluorescence measurements of PLs in the presence of ANS to test whether these proteins, following the exposure of mussels to mercury and chromium, had undergone conformational changes. So, we evaluated whether there were alterations in the fluorescence intensity of PLs of exposed mussels.

**Figure 7:**
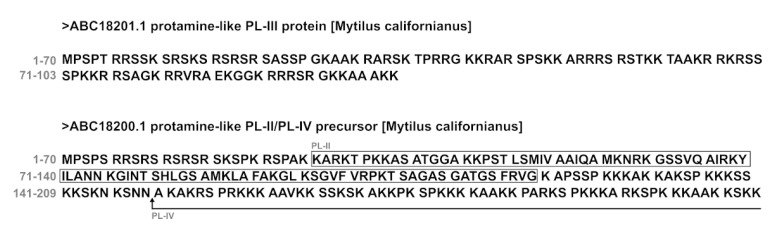
Complete aminoacidic sequences of PL-III and PL-II/PL-IV from *M. californianus* [50]. The box in the figure represents the amino acids of PL-II. From the arrow the amino acids sequence of PL-IV is indicated.

As a matter of fact, it is just at the highest doses of exposure, both to chromium and mercury, that we observed the greatest alterations in the fluorescence measurements of PLs compared to those from unexposed mussels. PLs are extremely basic proteins, particularly rich in Arginine. Arginine is a cationic amino acid, essential for ensuring strong interactions between the negatively charged surface of bacteria. This amino acid can interact both electrostatically and by forming hydrogen bonds with the negatively charged phospholipids of the bacterial cell membrane [30,31]. It is well known that positive charges are essential for antibacterial activity because they allow protein–membrane interactions, but at the same time, they represent a steric encumbrance for crossing the bacterial membrane [32,33]. The increased fluorescence of PLs observed under the conditions of exposure to the highest concentrations (100 pM HgCl_2_ and 100 nM Cr(VI)) could be responsible for the higher MIC and MBC values obtained for the PLs of mussels in these conditions. This because the increase in PLs fluorescence is compatible with a conformational change of PLs after the mussels’ exposure to mercury and chromium, leading to a different arrangement of positively charged amino acids, and consequently, a different interaction of PLs with the bacterial membrane. This results in a lower antibacterial capacity of these proteins, compared to other exposure conditions. In support of this, Pearson’s correlations confirm a direct implication of heavy metals’ concentrations on observed PLs changes in MIC and MBC values, which can be explained by the conformational changes that metals have induced, as has been described. Our results are in line with those reported by Wang et al., 2021 [34]. These authors demonstrated that modifications relative to the structure of antimicrobial peptides promote a decrease in their antibacterial activity. Furthermore, the antibacterial efficacy of other molecules, such as DMHNHC14-rich vesicles against Gram-positive bacteria, has been reduced as a response to a decrease in cationic surfactant content [35]. In addition, we should not forget that hydrophobicity is also critical for good antimicrobial activity, as reported by Kumar et al. (2018) [36], and it has also been shown that the increased hydrophobicity of arginine-rich peptides promotes entry into cells through the formation of membranous particles on the cell surface [37]. The variations of hydrophobicity directly alter the antimicrobial activity of AMP. In our study, we conducted the fluorescence analyses by using the 8-anilino-1-naphthalenesulfonic acid (ANS) for the absence of aromatic amino acids in the PLs. ANS, as an extrinsic fluorescent probe, is widely utilized to characterize proteins in various states [38]. Enhancement of ANS fluorescence indicates that the hydrophobic clusters of proteins are exposed [39,40]. The increased fluorescence of ANS observed with the PLs of mussels exposed to the highest doses of the two heavy metals tested in this work could explain their reduced antibacterial activity. On the other hand, just in *M. galloprovincialis*, Gorinstein et al., 2005 [41] reported a partial unfolding of proteins extracted from soft tissues in mussels collected from polluted areas, and these proteins showed an increase in fluorescence intensity and hydrophobicity. That proper hydrophobicity is very important for antibacterial activity, which is also confirmed by the work of Chen et al., 2007 [42]. These researchers examined the influence of the hydrophobicity of a synthetic α helical AMP on antimicrobial activity [42], reaching the conclusion that there is an optimal hydrophobicity required for good antimicrobial activity. Sequences with hydrophobicity below and far above this threshold rendered the peptides inactive [42]. After all, PLs tend to form aggregates, for example, PL-III, which is the most abundant of the three PLs and tends to form tetramers [43,44]. The sperm nuclear basic protein composition has been thoroughly studied and shown to be extremely conserved in all the species of Mytilus that have been studied so far and which include: *galloprovincialis, edulis, califotrnianuus, and trossulus* [45,46,47,48,49,50]. The following are the complete aminoacidic sequences of PL-III and PL-II/PL-IV from *M. californianus* (Figure 7) [50].

In order to understand which regions in these proteins (for example, hydrophobic regions responsible for antibacterial activity) can be damaged when interacting with heavy metals, we provided the aminoacidic sequence of the PL proteins of *Mytilus californianus*.

**Figure 8:**
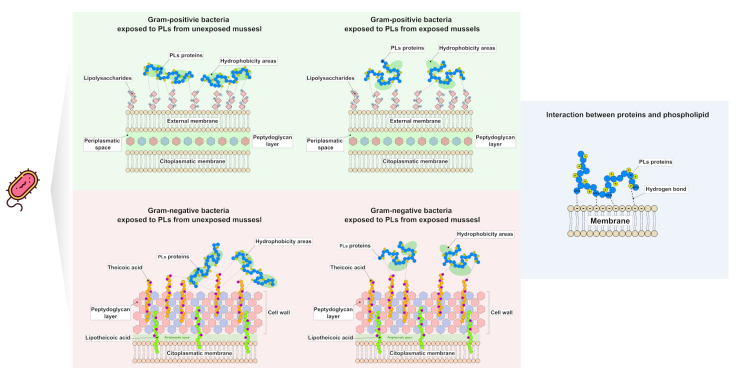
Hypothetical molecular models that explain how the binding of PLs to the membrane of Gram-positive and Gram-negative bacteria changes following the exposure of mussels to heavy metals. Hypothetical molecular models explaining how PLs from mussels exposed to the heavy metals tested bind differently to the membrane of Gram-positive and Gram-negative bacteria.

In light of all these considerations, based on our results, we can hypothesize the existence of potential molecular mechanisms that could explain the reduction in the antibacterial activity of PLs extracted from mussels exposed to these heavy metals. As reported in Figure 8, a different arrangement of positive charges on the proteins’ surfaces, induced by metals, results in a different interaction between PLs and an available negative charge on the bacterial surface, which probably prevents the penetration of PLs inside microbial cells and reduces the antibacterial features.

**Figure 9:**
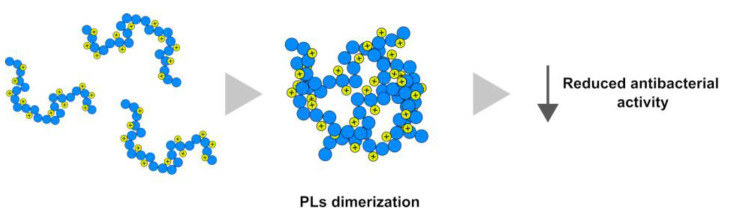
The dimerization of PLs causes the reduction in antibacterial activity.

This hypothesis, of course, assumes that there is a difference in exposed hydrophobic regions between the PLs of mussels not exposed to metals and exposed to chromium or mercury. Taking into account that optimal hydrophobicity is required for good antimicrobial activity; these differences could explain the reduced antibacterial activity of these proteins. The protamine-like proteins, as all the sperm nuclear basic proteins, have a natural tendency to form aggregates [51] for their function. The fact that the possible aggregates are not visible on SDS-PAGE or AU-PAGE is due to denaturing conditions, but the fact that these proteins form self-aggregates cannot be ruled out. Therefore, another hypothesis that explains the decrease in the antibacterial activity of PLs, always linked to the increase in hydrophobicity, could also be the increased likelihood of dimerization, which prevents access of the peptide to the bacterial membrane, as shown in Figure 9.

**Figure 10:**
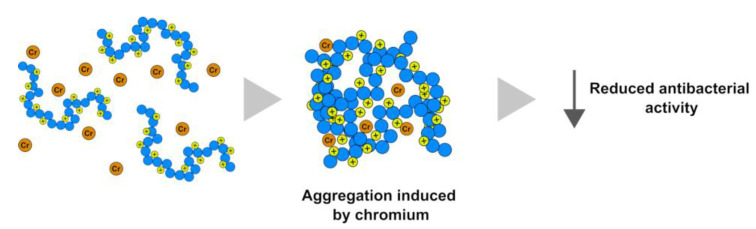
PL–chromium complexes have reduced antibacterial activity compared to PLs.

Another explanation for the reduced antibacterial activity of PLs from exposed mussels could depend, at least for chromium, on the possible complexes formed between PLs and chromium. In fact, it has been observed that the MIC values of some chromium complexes containing amino acids, such as ligands against Gram-negative and Gram-positive bacteria, are higher than the values of the standard antibiotic (Figure 10) [52].

However, how do these metals reach the PLs that determine their conformational changes? The answer: through the gonads. In fact, though these tissues are not used for filtration, they demonstrated that they have a comparable accumulation capacity of metals with respect to gills and digestive glands [17]. We have observed that when mussels are exposed to metals, the amounts of metal accumulated in sperm and PLs are generally very similar, suggesting that the metal accumulating in PLs is responsible for the metal accumulated in sperm [17]. We have already demonstrated that mercury accumulates in the gonads of mussels that are exposed to this metal [19], and that chromium does as well (manuscript in preparation). These are the only possible hypotheses that can be obtained with the data we have at present. In addition, we can certainly rule out the possibility that the reduction in the antibacterial activity of PLs in the mussels exposed to the metals tested in this work can be attributed to the loss of a fraction of the proteins due to thermolability, as PLs are extremely thermostable proteins; in fact, the result of treating these proteins under various experimental conditions at 37 °C for 18 h shows the integrity of PLs (Figure 6). Of course, our findings represent the first step in relating the antibacterial efficacy of PLs to their molecular changes. These considerations imply that further studies are required to explore the possible molecular sites involved in this process in greater detail. Therefore, our findings simply represent one piece of a large puzzle that everyone would like to complete. In particular, as a future perspective, we plan to label the PLs of exposed mussels with a fluorophore and make them interact with bacteria to evaluate whether there are differences in the mechanism of action between the PLs from unexposed and exposed mussels. At the present, we labeled PLs with a fluorophore (DEAC, SE) and analyzed their ability to interact with bacterial cells. We conducted this assay only on *Enterococcus faecalis* ATCC 29212. Preliminary data show that the proteins both interact with the membrane and penetrate the bacterial cell. It is not possible to make a quantitative assessment using the fluorophore, but what we have observed from experiments that were conducted before is that, at least for mercury, PLs from mussels exposed to this metal have a reduced ability to bind DNA [19]. Since the antibacterial molecules that are able to enter the bacterial cell have the inhibition of the transcription process or protein synthesis as their mechanism, it could be hypothesized that the reduction of antibacterial activity may depend on the reduced ability of PLs to bind DNA and, presumably, RNA, as well due to the conformational change that these proteins undergo as a result of mussel exposure to these heavy metals. We will also attempt to validate the hypotheses made about the possible molecular mechanisms underlying the reduction in the antibacterial activity of these proteins when derived from mussels exposed to these metals. However, we should be aware of the fact that potential new natural molecules with antibacterial activity can undergo a reduction in their antibacterial activity due to the effect of environmental pollution. So, this is a rather unfortunate fact to take into account because when marine creatures provide new bioactive molecules, environmental pollution can reduce the potential of these molecules over the years. Nevertheless, we believe in the validity of the pioneering work of our study since our unprecedented results can stimulate further investigations on the antibacterial activity of PLs, also considering that antibacterial agents frequently exhibit a strong relation between their antibacterial capacities and antioxidant power [53].

## 4. Materials and Methods

### 4.1. The Ethics Statement

The research described herein was performed on the marine invertebrate *M. galloprovincialis* (Lamarck, 1819), which is not protected by any environmental agency in Italy. This study was conducted in strict accordance with European (Directive 2010/63) and Italian (Legislative Decree n. 116/1992) legislation on the care and use of animals for scientific purposes.

### 4.2. Materials and Animal Collection

Chemical reagents were obtained from Merk Life Science S.r.l. (Milan, Italy). Electrophoresis reagents and apparatus were from Bio-Rad (Bio-Rad Laboratories S.r.l., Milan, Italy). Adult mussels *M. galloprovincialis* were kindly provided by Eurofish Napoli S.r.l., Baia, Naples.

### 4.3. Treatment of Mussels and Spermatozoa Collection

Adult mussels *M. galloprovincialis* of different sexes, with an average shell length of 4.93 ± 0.17 cm, were supplied by Eurofish Napoli S.r.l. Baia, Naples and used in this investigation. Experiments were carried out during February and March, 2022. Fifteen mussels of unknown sex were exposed in laboratory plastic tanks for 24 h (36 cm × 22 cm × 22 cm), containing 7 L of 33 ‰ artificial sea water (ASW) with the following composition for 1 L: NaCl 29.2 g, KCl 0.60 g, MgCl_2_ 1.2 g, NaHCO_3_ 0.20 g, and CaCl_2_ 1.08 g. The exposure to heavy metals (1, 10, 100 HgCl_2_ and 1, 10, 100 nM Cr(VI)) was carried out at 18 ± 1 °C for 24 h, every 12 h, the ASW was changed; the dissolved oxygen and temperature were recorded at predefined time intervals. One tank with 15 mussels was prepared in only ASW (unexposed condition, control condition). After the exposure, mussels were opened, forcing the valves with a knife; the knife was used with care so as to not cut the soft tissue. Gametes were obtained by stimulation of the male gonads with a Pasteur pipette and seawater. The sex of the mussels was determined by examining the gametes under a light microscope. In brief, the semen collected from all the male mussels contained in the tanks corresponding to a specific condition was pooled and centrifuged at 1000× *g* for 2 min at 4 °C to remove debris. The semen was filtered through gauze, and then centrifuged at 200× *g* for 3 min at 4 °C. Male gametes were obtained by centrifuging the former supernatant at 9000× *g* for 10 min at 4 °C and collected in pellets of about 200 µL volume, as previously described [54].

### 4.4. Extraction and Purification of M. galloprovincialis PLs

Perchloric acid (PCA) at a final concentration of 5% was used for the extraction of PLs from spermatozoa of *M. galloprovincialis* unexposed and exposed to HgCl_2_ (1, 10, and 100 pM) and Cr(VI) (1, 10, and 100 nM), following the procedure described in [55]. We extracted to observe protamine-like proteins with PCA because this acid, in this concentration, assures the extraction of the exclusive protamine-like proteins and not the histone core, which represent the other basic protein component of the sperm chromatin of these mussels. In brief, PL proteins were extracted from 10 sperm pellets, corresponding to the exposure of mussels to a specific concentration of pM HgCl_2_ and nM Cr(VI) and from the spermatozoa of unexposed mussels using 5% (PCA). Spermatozoa pellets were homogenized in a potter with 15 mL of distilled water, and then, acid extraction with PCA was performed adding PCA for a final concentration of 5%. The sample containing PCA-soluble PL proteins was then extensively dialyzed against distilled water in order to remove all PCA and then lyophilized and stored at −80 °C.

### 4.5. Electrophoretic Analyses on Polyacryilamide Gels of PLs from Unexposed and Exposed M. galloprovincialis

Protein samples were analyzed using two different electrophoretic analysis methods: AU-PAGE, as previously described by Piscopo et al., 2018 [13], with minor modifications, and SDS-PAGE, as described below. The gel recipe for urea-acetic acid-polyacrylamide gel electrophoresis (AU-PAGE) was as follows: 15% acrylamide/bis-acrylamide (starting from a solution of acrylamide/bis-acrylamide, 30:0.2 ratio). In brief, the gel was prepared with 2.5 M urea, 5% acetic acid, 0.75% TEMED, and 0.15% APS. After approximately 30 min of polymerization, a pre-run was carried out for 1 h at 150 V in 5% acetic acid running buffer. Before the run, the protein samples were denatured at room temperature for 30 min in a 8 M urea solution. A total of 4 µg of protein was loaded, and the electrophoretic run was conducted at 120 V for approximately 1 h. Regarding SDS-PAGE, the electrophoretic run was performed using a 4–20% Tris-Glycine 1.0 mm gradient gel from Thermo Fisher Novex, Waltham, MA, USA. A total of 4 µg of samples was boiled at 100 °C for 10 min with 10 µL of 1X Laemmli buffer. The electrophoretic run was performed in Tris Glycine 2X running buffer at 150 V for about 1 h. The gel was stained both with a solution of 0.25% Blue Comassie in 30% methanol and 10% acetic acid. Finally, the image was acquired with the GelDoc Biorad system, using the ImageLab 6.0.1 (build 34) software program (BioRad, Hercules, CA, USA).

### 4.6. Thermal Stability of PLs

PLs from unexposed and exposed mussel were used to test their thermal stability. Briefly, 10 µg of PLs of all conditions was placed at 37 °C for 18 h in sterilized water and subsequently analyzed in SDS-PAGE 4–20% Tris-Glycine 1.0 mm gradient gel (Thermo Fisher Novex).

### 4.7. Antibacterial Activity of M. galloprovincialis PLs

The evaluation of the antimicrobial activity of *M. galloprovincialis* PLs was performed using the broth dilution method (MH broth), as previously described in Verrillo et al., 2021; 2022; 2023 [53,56,57], with a minor modification. The bacterial strains used in this study included *Escherichia coli* ATCC 35218, *Pseudomonas aeruginosa* ATCC 27355, *Klebsiella pneumoniae* ATCC 700503, *Staphylococcus aureus* ATCC 5538P, and *Enterococcus faecalis* ATCC 29212 (Table 5). Briefly, bacterial strains were grown on Mueller Hinton (MH) agar plates (DIFCO) and suspended in Mueller Hinton (MH) broth (DIFCO). The bacterial broth culture was incubated at 37 °C until it achieved or exceeded the turbidity of the 0.5 McFarland standard. Then, all bacterial suspensions containing 10^6^ cells/mL were carried out, diluting the previous broth culture at 1:100, and ten serial two-fold dilutions were performed for the several PL concentrations in a range between 1–1000 μg/mL in 0.05 M Tris buffer (pH 7.4). A different solution of tetracycline and ampicillin was used as positive control. The combination of these two antibiotics has a wide range of antimicrobial activity against Gram-positive and Gram-negative bacteria. The bacterial suspensions were aerobically incubated for 18 ± 1 h at 37 °C. MIC values were estimated measuring the absorbance of microtiter plates at 570 nm. The lowest concentration at which no turbidity was observed was considered the MIC value. Three independent experiments were performed for each MIC test. Additionally, MBC was then estimated as the lowest concentration of the bactericidal molecule resulting in no growth.

### 4.8. Fluorescence Measurements of M. galloprovincialis PLs

Fluorescence measurements of PLs from unexposed and exposed mussels to HgCl_2_ (1, 10, and 100 pM) and Cr(VI) (1, 10, and 100 nM) were conducted in a 1 cm optical path cuvette using a PerkinElmer luminescence spectrometer LS-55 and 1 mL of PL solution 1 mg/mL in water in the presence of 5 µM 8-anilino-1-naphthalenesulfonic acid (ANS), as an extrinsic fluorescent probe. Fluorescence spectra in the emission region of wavelengths 410 to 600 nm were obtained after excitation at 350 nm. The data were analyzed with GraphPad Prism (v. 9.5.1.733).

### 4.9. Statistical Analysis

The data were analyzed by two-way ANOVA followed by Tukey’s test for MIC and MBC data. Values were considered significant when *p* < 0.05. Statistically significant differences are defined at the 95% confidence interval of the MIC and MBC data. In additional, the correlation analysis was performed using a Pearson coefficient between the concentration of heavy metals used and the bacterial strains. The analyses were performed with GraphPad Prism (v. 9.5.1.733).

## Figures and Tables

**Table 1:** Pearson’s correlation between HgCl_2_ concentrations and MIC values obtained by PLs extracted from mussels exposed to HgCl_2_. ns = not significance; * = *p*-value ≤ 0.05; ** = *p*-value ≤ 0.01; **** = *p*-value ≤ 0.001.

	*E. coli* ATCC 35218	*P. aeruginosa* ATCC 27355	*K. pneumoniae* ATCC 700503	*S. aureus* ATCC 5538P	*E. faecalis* ATCC 29212
r	0.9999	0.9934	0.9476	0.9617	0.9116
95% confidence interval	0.9971 to 1.000	0.7140 to 0.9999	−0.1508 to 0.9989	0.007551 to 0.9992	−0.3993 to 0.9982
R-squared	0.9999	0.9868	0.8980	0.9248	0.8311
*p* (two-tailed)	<0.0001	0.0066	0.0524	0.0383	0.0884
*p* value summary	****	**	ns	*	ns
Significant? (alpha = 0.05)	Yes	Yes	No	Yes	No

**Table 2:** Pearson’s correlation between HgCl_2_ concentrations and MBC values obtained by PLs extracted from mussels exposed to HgCl_2_. ns = not significance; * = *p*-value ≤ 0.05; ** = *p*-value ≤ 0.01; **** = *p*-value ≤ 0.001.

	*E. coli* ATCC 35218	*P. aeruginosa* ATCC 27355	*K. pneumoniae* ATCC 700503	*S. aureus* ATCC 5538P	*E. faecalis* ATCC 29212
r	1.000	0.9927	0.9614	0.9577	0.9120
95% confidence interval	0.9987 to 1.000	0.6866 to 0.9999	0.003564 to 0.9992	−0.04278 to 0.9991	−0.3975 to 0.9982
R-squared	0.9999	0.9854	0.9242	0.9172	0.8317
*p* (two-tailed)	<0.0001	0.0073	0.0386	0.0423	0.0880
*p* value summary	****	**	*	*	ns
Significant? (alpha = 0.05)	Yes	Yes	Yes	Yes	No

**Table 3:** Pearson’s correlation between Cr(VI) concentrations and MIC values obtained by PLs extracted from mussels exposed to Cr(VI). ns = not significance; * = *p*-value ≤ 0.05; ** = *p*-value ≤ 0.01; *** = *p*-value ≤ 0.001.

	*E. coli* ATCC 35218	*P. aeruginosa* ATCC 27355	*K. pneumoniae* ATCC 700503	*S. aureus* ATCC 5538P	*E. faecalis* ATCC 29212
r	0.9993	0.9735	0.9888	0.9934	0.9364
95% confidence interval	0.9669 to 1.000	0.1931 to 0.9995	0.5578 to 0.9998	0.7149 to 0.9999	−0.2466 to 0.9987
R-squared	0.9987	0.9477	0.9777	0.9869	0.8769
*p* (two-tailed)	0.0007	0.0265	0.0112	0.0066	0.0636
*p* value summary	***	*	*	**	ns
Significant? (alpha = 0.05)	Yes	Yes	Yes	Yes	No

**Table 4:** Pearson’s correlation between Cr(VI) concentrations and MBC values obtained by PLs extracted from mussels exposed to Cr(VI). ns = not significance; * = *p*-value ≤ 0.05; ** = *p*-value ≤ 0.01.

	*E. coli* ATCC 35218	*P. aeruginosa* ATCC 27355	*K. pneumoniae* ATCC 700503	*S. aureus* ATCC 5538P	*E. faecalis* ATCC 29212
r	0.9987	0.9954	0.9867	0.9953	0.9142
95% confidence interval	0.9379 to 1.000	0.7937 to 0.9999	0.4959 to 0.9997	0.7874 to 0.9999	−0.3863 to 0.9982
R-squared	0.9975	0.9909	0.9736	0.9906	0.8358
*p* (two-tailed)	0.0013	0.0046	0.0133	0.0047	0.0858
*p* value summary	**	**	*	**	ns
Significant? (alpha = 0.05)	Yes	Yes	Yes	Yes	No

**Table 5:** Bacteria strains and Gram-stained.

Bacteria Strains	Gram Stain
*Escherichia coli* ATCC 35218	Gram−
*Pseudomonas aeruginosa* ATCC 27355	Gram−
*Klebsiella pneumoniae* ATCC 700503	Gram−
*Staphylococcus aureus* ATCC 5538P	Gram+
*Enterococcus faecalis* ATCC 29212	Gram+

## Data Availability

Not applicable.

## Supplementary Materials

The following supporting information can be downloaded at: https://www.mdpi.com/article/10.3390/ijms24119345/s1.
