# Supplementary material for: Possible Molecular Mechanisms Underlying the Decrease in the Antibacterial Activity of Protamine-like Proteins after Exposure of Mytilus galloprovincialis to Chromium and Mercury
Source: Int J Mol Sci. 2023 May 26;24(11):9345. doi: 10.3390/ijms24119345 (PMC10253609; doi:10.3390/ijms24119345)
Supplement: Supplementary file 1 [file ijms-24-09345-s001.zip › ijms-2398628-supplementary.pdf]

Possible Molecular Mechanisms Underlying the Decrease in the Antibacterial Activity of Protamine-like Proteins after Exposure of *Mytilus galloprovincialis* to Chromium and Mercury

Carmela Marinaro [1,†], Gennaro Lettieri [1,†], Mariavittoria Verrillo [2], Michela Morelli [1], Federica Carraturo [1], Marco Guida [1] and Marina Piscopo [1,*]

[1] Department of Biology, University of Naples Federico II, 80126 Napoli, Italy; marco.guida@unina.it (M.G.)
[2] Department of Agricultural Sciences, Interdepartmental Research Centre of Nuclear Magnetic Resonance for the Environment, AgriFood and New Materials (CERMANU), University of Naples Federico II, 80055 Portici, Italy; mariavittoria.verrillo@unina.it
* Correspondence: marina.piscopo@unina.it
† These authors contributed equally to this work.

Multiple comparisons performed with Tukey's test following two-way ANOVA on $HgCl_2$ MIC. ns = not significance; **** = p-value ≤ 0.001.

Tukey's multiple comparisons test	Mean Diff.	95.00% CI of diff.	Summary	Adjusted P Value
***Escherichia coli* ATCC 35218**				
Unexposed vs. 1 pM Hg	-0.30	-1.773 to 1.173	ns	0.9907
Unexposed vs. 10 pM Hg	-5.13	-6.607 to -3.660	****	<0.0001
Unexposed vs. 100 pM Hg	-47.90	-49.37 to -46.43	****	<0.0001
Unexposed vs. Ampicilline	26.90	25.43 to 28.37	****	<0.0001
Unexposed vs. Tetracicline	26.50	25.03 to 27.97	****	<0.0001
1 pM Hg vs. 10 pM Hg	-4.83	-6.307 to -3.360	****	<0.0001
1 pM Hg vs. 100 pM Hg	-47.60	-49.07 to -46.13	****	<0.0001
1 pM Hg vs. Ampicilline	27.20	25.73 to 28.67	****	<0.0001
1 pM Hg vs. Tetracicline	26.80	25.33 to 28.27	****	<0.0001
10 pM Hg vs. 100 pM Hg	-42.77	-44.24 to -41.29	****	<0.0001
10 pM Hg vs. Ampicilline	32.03	30.56 to 33.51	****	<0.0001
10 pM Hg vs. Tetracicline	31.63	30.16 to 33.11	****	<0.0001
100 pM Hg vs. Ampicilline	74.80	73.33 to 76.27	****	<0.0001
100 pM Hg vs. Tetracicline	74.40	72.93 to 75.87	****	<0.0001
Ampicilline vs. Tetracicline	-0.40	-1.873 to 1.073	ns	0.9666
***Pseudomonas aeruginosa* ATCC 27355**				
Unexposed vs. 1 pM Hg	-1.07	-2.540 to 0.4065	ns	0.2856
Unexposed vs. 10 pM Hg	-6.93	-8.407 to -5.460	****	<0.0001
Unexposed vs. 100 pM Hg	-31.57	-33.04 to -30.09	****	<0.0001
Unexposed vs. Ampicilline	22.93	21.46 to 24.41	****	<0.0001
Unexposed vs. Tetracicline	22.67	21.19 to 24.14	****	<0.0001
1 pM Hg vs. 10 pM Hg	-5.87	-7.340 to -4.393	****	<0.0001
1 pM Hg vs. 100 pM Hg	-30.50	-31.97 to -29.03	****	<0.0001
1 pM Hg vs. Ampicilline	24.00	22.53 to 25.47	****	<0.0001
1 pM Hg vs. Tetracicline	23.73	22.26 to 25.21	****	<0.0001
10 pM Hg vs. 100 pM Hg	-24.63	-26.11 to -23.16	****	<0.0001
10 pM Hg vs. Ampicilline	29.87	28.39 to 31.34	****	<0.0001
10 pM Hg vs. Tetracicline	29.60	28.13 to 31.07	****	<0.0001
100 pM Hg vs. Ampicilline	54.50	53.03 to 55.97	****	<0.0001
100 pM Hg vs. Tetracicline	54.23	52.76 to 55.71	****	<0.0001
Ampicilline vs. Tetracicline	-0.27	-1.740 to 1.207	ns	0.9946
***Klebsiella pneumoniae* ATCC 700503**				
Unexposed vs. 1 pM Hg	-2.80	-4.273 to -1.327	****	<0.0001
Unexposed vs. 10 pM Hg	-6.87	-8.340 to -5.393	****	<0.0001
Unexposed vs. 100 pM Hg	-16.17	-17.64 to -14.69	****	<0.0001
Unexposed vs. Ampicilline	23.97	22.49 to 25.44	****	<0.0001
Unexposed vs. Tetracicline	24.37	22.89 to 25.84	****	<0.0001
1 pM Hg vs. 10 pM Hg	-4.07	-5.540 to -2.593	****	<0.0001
1 pM Hg vs. 100 pM Hg	-13.37	-14.84 to -11.89	****	<0.0001

1 pM Hg vs. Ampicilline	26.77	25.29 to 28.24	****	<0.0001
1 pM Hg vs. Tetracicline	27.17	25.69 to 28.64	****	<0.0001
10 pM Hg vs. 100 pM Hg	-9.30	-10.77 to -7.827	****	<0.0001
10 pM Hg vs. Ampicilline	30.83	29.36 to 32.31	****	<0.0001
10 pM Hg vs. Tetracicline	31.23	29.76 to 32.71	****	<0.0001
100 pM Hg vs. Ampicilline	40.13	38.66 to 41.61	****	<0.0001
100 pM Hg vs. Tetracicline	40.53	39.06 to 42.01	****	<0.0001
Ampicilline vs. Tetracicline	0.40	-1.073 to 1.873	ns	0.9666
***Staphylococcus aureus* ATCC 5538P**				
Unexposed vs. 1 pM Hg	-3.93	-5.407 to -2.460	****	<0.0001
Unexposed vs. 10 pM Hg	-6.80	-8.273 to -5.327	****	<0.0001
Unexposed vs. 100 pM Hg	-18.63	-20.11 to -17.16	****	<0.0001
Unexposed vs. Ampicilline	23.07	21.59 to 24.54	****	<0.0001
Unexposed vs. Tetracicline	23.60	22.13 to 25.07	****	<0.0001
1 pM Hg vs. 10 pM Hg	-2.87	-4.340 to -1.393	****	<0.0001
1 pM Hg vs. 100 pM Hg	-14.70	-16.17 to -13.23	****	<0.0001
1 pM Hg vs. Ampicilline	27.00	25.53 to 28.47	****	<0.0001
1 pM Hg vs. Tetracicline	27.53	26.06 to 29.01	****	<0.0001
10 pM Hg vs. 100 pM Hg	-11.83	-13.31 to -10.36	****	<0.0001
10 pM Hg vs. Ampicilline	29.87	28.39 to 31.34	****	<0.0001
10 pM Hg vs. Tetracicline	30.40	28.93 to 31.87	****	<0.0001
100 pM Hg vs. Ampicilline	41.70	40.23 to 43.17	****	<0.0001
100 pM Hg vs. Tetracicline	42.23	40.76 to 43.71	****	<0.0001
Ampicilline vs. Tetracicline	0.53	-0.9399 to 2.007	ns	0.8929
***Enterococcus faecalis* ATCC 29212**				
Unexposed vs. 1 pM Hg	-4.10	-5.573 to -2.627	****	<0.0001
Unexposed vs. 10 pM Hg	-8.10	-9.573 to -6.627	****	<0.0001
Unexposed vs. 100 pM Hg	-16.00	-17.47 to -14.53	****	<0.0001
Unexposed vs. Ampicilline	21.03	19.56 to 22.51	****	<0.0001
Unexposed vs. Tetracicline	21.50	20.03 to 22.97	****	<0.0001
1 pM Hg vs. 10 pM Hg	-4.00	-5.473 to -2.527	****	<0.0001
1 pM Hg vs. 100 pM Hg	-11.90	-13.37 to -10.43	****	<0.0001
1 pM Hg vs. Ampicilline	25.13	23.66 to 26.61	****	<0.0001
1 pM Hg vs. Tetracicline	25.60	24.13 to 27.07	****	<0.0001
10 pM Hg vs. 100 pM Hg	-7.90	-9.373 to -6.427	****	<0.0001
10 pM Hg vs. Ampicilline	29.13	27.66 to 30.61	****	<0.0001
10 pM Hg vs. Tetracicline	29.60	28.13 to 31.07	****	<0.0001
100 pM Hg vs. Ampicilline	37.03	35.56 to 38.51	****	<0.0001
100 pM Hg vs. Tetracicline	37.50	36.03 to 38.97	****	<0.0001
Ampicilline vs. Tetracicline	0.47	-1.007 to 1.940	ns	0.9365

Multiple comparisons performed with Tukey's test following two-way ANOVA on Cr(VI) MIC. ns = not significance; * = p-value ≤ 0.05; **** = p-value ≤ 0.001.

Tukey's multiple comparisons test	Mean Diff.	95.00% CI of diff.	Summary	Adjusted P Value
**Escherichia coli ATCC 35218**				
Unexposed vs. 1 nM Cr(VI)	-0.567	-1.492 to 0.3591	ns	0.4723
Unexposed vs. 10 nM Cr(VI)	-3.1	-4.026 to -2.174	****	<0.0001
Unexposed vs. 100 nM Cr(VI)	-49	-49.93 to -48.07	****	<0.0001
Unexposed vs. Ampicilline	28.27	27.34 to 29.19	****	<0.0001
Unexposed vs. Tetracicline	27.97	27.04 to 28.89	****	<0.0001
1 nM Cr(VI) vs. 10 nM Cr(VI)	-2.533	-3.459 to -1.608	****	<0.0001
1 nM Cr(VI) vs. 100 nM Cr(VI)	-48.43	-49.36 to -47.51	****	<0.0001
1 nM Cr(VI) vs. Ampicilline	28.83	27.91 to 29.76	****	<0.0001
1 nM Cr(VI) vs. Tetracicline	28.53	27.61 to 29.46	****	<0.0001
10 nM Cr(VI) vs. 100 nM Cr(VI)	-45.9	-46.83 to -44.97	****	<0.0001
10 nM Cr(VI) vs. Ampicilline	31.37	30.44 to 32.29	****	<0.0001
10 nM Cr(VI) vs. Tetracicline	31.07	30.14 to 31.99	****	<0.0001
100 nM Cr(VI) vs. Ampicilline	77.27	76.34 to 78.19	****	<0.0001
100 nM Cr(VI) vs. Tetracicline	76.97	76.04 to 77.89	****	<0.0001
Ampicilline vs. Tetracicline	-0.3	-1.226 to 0.6257	ns	0.9304
***Pseudomonas aeruginosa* ATCC 27355**				
Unexposed vs. 1 nM Cr(VI)	-0.933	-1.859 to -0.007608	*	0.047
Unexposed vs. 10 nM Cr(VI)	-6.433	-7.359 to -5.508	****	<0.0001
Unexposed vs. 100 nM Cr(VI)	-19.33	-20.26 to -18.41	****	<0.0001
Unexposed vs. Ampicilline	24	23.07 to 24.93	****	<0.0001
Unexposed vs. Tetracicline	23.63	22.71 to 24.56	****	<0.0001
1 nM Cr(VI) vs. 10 nM Cr(VI)	-5.5	-6.426 to -4.574	****	<0.0001
1 nM Cr(VI) vs. 100 nM Cr(VI)	-18.4	-19.33 to -17.47	****	<0.0001
1 nM Cr(VI) vs. Ampicilline	24.93	24.01 to 25.86	****	<0.0001
1 nM Cr(VI) vs. Tetracicline	24.57	23.64 to 25.49	****	<0.0001
10 nM Cr(VI) vs. 100 nM Cr(VI)	-12.9	-13.83 to -11.97	****	<0.0001
10 nM Cr(VI) vs. Ampicilline	30.43	29.51 to 31.36	****	<0.0001
10 nM Cr(VI) vs. Tetracicline	30.07	29.14 to 30.99	****	<0.0001
100 nM Cr(VI) vs. Ampicilline	43.33	42.41 to 44.26	****	<0.0001
100 nM Cr(VI) vs. Tetracicline	42.97	42.04 to 43.89	****	<0.0001
Ampicilline vs. Tetracicline	-0.367	-1.292 to 0.5591	ns	0.851
***Klebsiella pneumonia*e ATCC 700503**				
Unexposed vs. 1 nM Cr(VI)	-1.1	-2.026 to -0.1743	*	0.011
Unexposed vs. 10 nM Cr(VI)	-3.567	-4.492 to -2.641	****	<0.0001
Unexposed vs. 100 nM Cr(VI)	-13.9	-14.83 to -12.97	****	<0.0001
Unexposed vs. Ampicilline	25	24.07 to 25.93	****	<0.0001
Unexposed vs. Tetracicline	24.97	24.04 to 25.89	****	<0.0001
1 nM Cr(VI) vs. 10 nM Cr(VI)	-2.467	-3.392 to -1.541	****	<0.0001

1 nM Cr(VI) vs. 100 nM Cr(VI)	-12.8	-13.73 to -11.87	****	<0.0001
1 nM Cr(VI) vs. Ampicilline	26.1	25.17 to 27.03	****	<0.0001
1 nM Cr(VI) vs. Tetracicline	26.07	25.14 to 26.99	****	<0.0001
10 nM Cr(VI) vs. 100 nM Cr(VI)	-10.33	-11.26 to -9.408	****	<0.0001
10 nM Cr(VI) vs. Ampicilline	28.57	27.64 to 29.49	****	<0.0001
10 nM Cr(VI) vs. Tetracicline	28.53	27.61 to 29.46	****	<0.0001
100 nM Cr(VI) vs. Ampicilline	38.9	37.97 to 39.83	****	<0.0001
100 nM Cr(VI) vs. Tetracicline	38.87	37.94 to 39.79	****	<0.0001
Ampicilline vs. Tetracicline	-0.033	-0.9591 to 0.8924	ns	>0.9999
***Staphylococcus aureus* ATCC 5538P**				
Unexposed vs. 1 nM Cr(VI)	-1.033	-1.959 to -0.1076	*	0.0201
Unexposed vs. 10 nM Cr(VI)	-4.1	-5.026 to -3.174	****	<0.0001
Unexposed vs. 100 nM Cr(VI)	-18.37	-19.29 to -17.44	****	<0.0001
Unexposed vs. Ampicilline	21.63	20.71 to 22.56	****	<0.0001
Unexposed vs. Tetracicline	21.63	20.71 to 22.56	****	<0.0001
1 nM Cr(VI) vs. 10 nM Cr(VI)	-3.067	-3.992 to -2.141	****	<0.0001
1 nM Cr(VI) vs. 100 nM Cr(VI)	-17.33	-18.26 to -16.41	****	<0.0001
1 nM Cr(VI) vs. Ampicilline	22.67	21.74 to 23.59	****	<0.0001
1 nM Cr(VI) vs. Tetracicline	22.67	21.74 to 23.59	****	<0.0001
10 nM Cr(VI) vs. 100 nM Cr(VI)	-14.27	-15.19 to -13.34	****	<0.0001
10 nM Cr(VI) vs. Ampicilline	25.73	24.81 to 26.66	****	<0.0001
10 nM Cr(VI) vs. Tetracicline	25.73	24.81 to 26.66	****	<0.0001
100 nM Cr(VI) vs. Ampicilline	40	39.07 to 40.93	****	<0.0001
100 nM Cr(VI) vs. Tetracicline	40	39.07 to 40.93	****	<0.0001
Ampicilline vs. Tetracicline	0	-0.9257 to 0.9257	ns	>0.9999
***Enterococcus faecalis* ATCC 29212**				
Unexposed vs. 1 nM Cr(VI)	-0.7	-1.626 to 0.2257	ns	0.2414
Unexposed vs. 10 nM Cr(VI)	-5.733	-6.659 to -4.808	****	<0.0001
Unexposed vs. 100 nM Cr(VI)	-12.67	-13.59 to -11.74	****	<0.0001
Unexposed vs. Ampicilline	22.37	21.44 to 23.29	****	<0.0001
Unexposed vs. Tetracicline	23.2	22.27 to 24.13	****	<0.0001
1 nM Cr(VI) vs. 10 nM Cr(VI)	-5.033	-5.959 to -4.108	****	<0.0001
1 nM Cr(VI) vs. 100 nM Cr(VI)	-11.97	-12.89 to -11.04	****	<0.0001
1 nM Cr(VI) vs. Ampicilline	23.07	22.14 to 23.99	****	<0.0001
1 nM Cr(VI) vs. Tetracicline	23.9	22.97 to 24.83	****	<0.0001
10 nM Cr(VI) vs. 100 nM Cr(VI)	-6.933	-7.859 to -6.008	****	<0.0001
10 nM Cr(VI) vs. Ampicilline	28.1	27.17 to 29.03	****	<0.0001
10 nM Cr(VI) vs. Tetracicline	28.93	28.01 to 29.86	****	<0.0001
100 nM Cr(VI) vs. Ampicilline	35.03	34.11 to 35.96	****	<0.0001
100 nM Cr(VI) vs. Tetracicline	35.87	34.94 to 36.79	****	<0.0001
Ampicilline vs. Tetracicline	0.8333	-0.09239 to 1.759	ns	0.1011

Multiple comparisons performed with Tukey's test following two-way ANOVA on $HgCl_2$ MBC. ns = not significance; * = p-value ≤ 0.05; **** = p-value ≤ 0.001.

Tukey's multiple comparisons test	Mean Diff.	95.00% CI of diff.	Summary	Adjusted P Value
***Escherichia coli* ATCC 35218**				
Unexposed vs. 1 pM Hg	-0.2	-1.694 to 1.294	ns	0.9987
Unexposed vs. 10 pM Hg	-4.867	-6.361 to -3.372	****	<0.0001
Unexposed vs. 100 pM Hg	-47.33	-48.83 to -45.84	****	<0.0001
Unexposed vs. Ampicilline	27.13	25.64 to 28.63	****	<0.0001
Unexposed vs. Tetracicline	27.13	25.64 to 28.63	****	<0.0001
1 pM Hg vs. 10 pM Hg	-4.667	-6.161 to -3.172	****	<0.0001
1 pM Hg vs. 100 pM Hg	-47.13	-48.63 to -45.64	****	<0.0001
1 pM Hg vs. Ampicilline	27.33	25.84 to 28.83	****	<0.0001
1 pM Hg vs. Tetracicline	27.33	25.84 to 28.83	****	<0.0001
10 pM Hg vs. 100 pM Hg	-42.47	-43.96 to -40.97	****	<0.0001
10 pM Hg vs. Ampicilline	32	30.51 to 33.49	****	<0.0001
10 pM Hg vs. Tetracicline	32	30.51 to 33.49	****	<0.0001
100 pM Hg vs. Ampicilline	74.47	72.97 to 75.96	****	<0.0001
100 pM Hg vs. Tetracicline	74.47	72.97 to 75.96	****	<0.0001
Ampicilline vs. Tetracicline	0	-1.494 to 1.494	ns	>0.9999
***Pseudomonas aeruginosa* ATCC 27355**				
Unexposed vs. 1 pM Hg	-1.667	-3.161 to -0.1725	*	0.0203
Unexposed vs. 10 pM Hg	-7.233	-8.728 to -5.739	****	<0.0001
Unexposed vs. 100 pM Hg	-31.57	-33.06 to -30.07	****	<0.0001
Unexposed vs. Ampicilline	23.2	21.71 to 24.69	****	<0.0001
Unexposed vs. Tetracicline	22.93	21.44 to 24.43	****	<0.0001
1 pM Hg vs. 10 pM Hg	-5.567	-7.061 to -4.072	****	<0.0001
1 pM Hg vs. 100 pM Hg	-29.9	-31.39 to -28.41	****	<0.0001
1 pM Hg vs. Ampicilline	24.87	23.37 to 26.36	****	<0.0001
1 pM Hg vs. Tetracicline	24.6	23.11 to 26.09	****	<0.0001
10 pM Hg vs. 100 pM Hg	-24.33	-25.83 to -22.84	****	<0.0001
10 pM Hg vs. Ampicilline	30.43	28.94 to 31.93	****	<0.0001
10 pM Hg vs. Tetracicline	30.17	28.67 to 31.66	****	<0.0001
100 pM Hg vs. Ampicilline	54.77	53.27 to 56.26	****	<0.0001
100 pM Hg vs. Tetracicline	54.5	53.01 to 55.99	****	<0.0001
Ampicilline vs. Tetracicline	-0.267	-1.761 to 1.228	ns	0.9949
***Klebsiella pneumoniae* ATCC 700503**				
Unexposed vs. 1 pM Hg	-2.567	-4.061 to -1.072	****	<0.0001
Unexposed vs. 10 pM Hg	-6.5	-7.994 to -5.006	****	<0.0001
Unexposed vs. 100 pM Hg	-16.97	-18.46 to -15.47	****	<0.0001
Unexposed vs. Ampicilline	24.43	22.94 to 25.93	****	<0.0001
Unexposed vs. Tetracicline	24.77	23.27 to 26.26	****	<0.0001
1 pM Hg vs. 10 pM Hg	-3.933	-5.428 to -2.439	****	<0.0001
1 pM Hg vs. 100 pM Hg	-14.4	-15.89 to -12.91	****	<0.0001

1 pM Hg vs. Ampicilline	27	25.51 to 28.49	****	<0.0001
1 pM Hg vs. Tetracicline	27.33	25.84 to 28.83	****	<0.0001
10 pM Hg vs. 100 pM Hg	-10.47	-11.96 to -8.972	****	<0.0001
10 pM Hg vs. Ampicilline	30.93	29.44 to 32.43	****	<0.0001
10 pM Hg vs. Tetracicline	31.27	29.77 to 32.76	****	<0.0001
100 pM Hg vs. Ampicilline	41.4	39.91 to 42.89	****	<0.0001
100 pM Hg vs. Tetracicline	41.73	40.24 to 43.23	****	<0.0001
Ampicilline vs. Tetracicline	0.3333	-1.161 to 1.828	ns	0.9859
***Staphylococcus aureus* ATCC 5538P**				
Unexposed vs. 1 pM Hg	-4.033	-5.528 to -2.539	****	<0.0001
Unexposed vs. 10 pM Hg	-7.267	-8.761 to -5.772	****	<0.0001
Unexposed vs. 100 pM Hg	-19.07	-20.56 to -17.57	****	<0.0001
Unexposed vs. Ampicilline	23.7	22.21 to 25.19	****	<0.0001
Unexposed vs. Tetracicline	24.33	22.84 to 25.83	****	<0.0001
1 pM Hg vs. 10 pM Hg	-3.233	-4.728 to -1.739	****	<0.0001
1 pM Hg vs. 100 pM Hg	-15.03	-16.53 to -13.54	****	<0.0001
1 pM Hg vs. Ampicilline	27.73	26.24 to 29.23	****	<0.0001
1 pM Hg vs. Tetracicline	28.37	26.87 to 29.86	****	<0.0001
10 pM Hg vs. 100 pM Hg	-11.8	-13.29 to -10.31	****	<0.0001
10 pM Hg vs. Ampicilline	30.97	29.47 to 32.46	****	<0.0001
10 pM Hg vs. Tetracicline	31.6	30.11 to 33.09	****	<0.0001
100 pM Hg vs. Ampicilline	42.77	41.27 to 44.26	****	<0.0001
100 pM Hg vs. Tetracicline	43.4	41.91 to 44.89	****	<0.0001
Ampicilline vs. Tetracicline	0.6333	-0.8609 to 2.128	ns	0.8114
***Enterococcus faecalis* ATCC 29212**				
Unexposed vs. 1 pM Hg	-4.7	-6.194 to -3.206	****	<0.0001
Unexposed vs. 10 pM Hg	-8.1	-9.594 to -6.606	****	<0.0001
Unexposed vs. 100 pM Hg	-16.4	-17.89 to -14.91	****	<0.0001
Unexposed vs. Ampicilline	21.57	20.07 to 23.06	****	<0.0001
Unexposed vs. Tetracicline	22.07	20.57 to 23.56	****	<0.0001
1 pM Hg vs. 10 pM Hg	-3.4	-4.894 to -1.906	****	<0.0001
1 pM Hg vs. 100 pM Hg	-11.7	-13.19 to -10.21	****	<0.0001
1 pM Hg vs. Ampicilline	26.27	24.77 to 27.76	****	<0.0001
1 pM Hg vs. Tetracicline	26.77	25.27 to 28.26	****	<0.0001
10 pM Hg vs. 100 pM Hg	-8.3	-9.794 to -6.806	****	<0.0001
10 pM Hg vs. Ampicilline	29.67	28.17 to 31.16	****	<0.0001
10 pM Hg vs. Tetracicline	30.17	28.67 to 31.66	****	<0.0001
100 pM Hg vs. Ampicilline	37.97	36.47 to 39.46	****	<0.0001
100 pM Hg vs. Tetracicline	38.47	36.97 to 39.96	****	<0.0001
Ampicilline vs. Tetracicline	0.5	-0.9942 to 1.994	ns	0.9209

Multiple comparisons performed with Tukey's test following two-way ANOVA on Cr(VI) MBC. ns = not significance; * = p-value ≤ 0.05; ** = p-value ≤ 0.01; **** = p-value ≤ 0.001.

Tukey's multiple comparisons test	Mean Diff.	95.00% CI of diff.	Summary	Adjusted P Value
***Escherichia coli* ATCC 35218**				
Unexposed vs. 1 nM Cr(VI)	-3.067	-5.121 to -1.013	***	0.0006
Unexposed vs. 10 nM Cr(VI)	-4.933	-6.987 to -2.879	****	<0.0001
Unexposed vs. 100 nM Cr(VI)	-52.93	-54.99 to -50.88	****	<0.0001
Unexposed vs. Ampicilline	29.7	27.65 to 31.75	****	<0.0001
Unexposed vs. Tetracicline	29.63	27.58 to 31.69	****	<0.0001
1 nM Cr(VI) vs. 10 nM Cr(VI)	-1.867	-3.921 to 0.1874	ns	0.0955
1 nM Cr(VI) vs. 100 nM Cr(VI)	-49.87	-51.92 to -47.81	****	<0.0001
1 nM Cr(VI) vs. Ampicilline	32.77	30.71 to 34.82	****	<0.0001
1 nM Cr(VI) vs. Tetracicline	32.7	30.65 to 34.75	****	<0.0001
10 nM Cr(VI) vs. 100 nM Cr(VI)	-48	-50.05 to -45.95	****	<0.0001
10 nM Cr(VI) vs. Ampicilline	34.63	32.58 to 36.69	****	<0.0001
10 nM Cr(VI) vs. Tetracicline	34.57	32.51 to 36.62	****	<0.0001
100 nM Cr(VI) vs. Ampicilline	82.63	80.58 to 84.69	****	<0.0001
100 nM Cr(VI) vs. Tetracicline	82.57	80.51 to 84.62	****	<0.0001
Ampicilline vs. Tetracicline	-0.067	-2.121 to 1.987	ns	>0.9999
***Pseudomonas aeruginosa* ATCC 27355**				
Unexposed vs. 1 nM Cr(VI)	2.633	0.5792 to 4.687	**	0.0048
Unexposed vs. 10 nM Cr(VI)	-2.367	-4.421 to -0.3126	*	0.0149
Unexposed vs. 100 nM Cr(VI)	-26.6	-28.65 to -24.55	****	<0.0001
Unexposed vs. Ampicilline	28.47	26.41 to 30.52	****	<0.0001
Unexposed vs. Tetracicline	28.53	26.48 to 30.59	****	<0.0001
1 nM Cr(VI) vs. 10 nM Cr(VI)	-5	-7.054 to -2.946	****	<0.0001
1 nM Cr(VI) vs. 100 nM Cr(VI)	-29.23	-31.29 to -27.18	****	<0.0001
1 nM Cr(VI) vs. Ampicilline	25.83	23.78 to 27.89	****	<0.0001
1 nM Cr(VI) vs. Tetracicline	25.9	23.85 to 27.95	****	<0.0001
10 nM Cr(VI) vs. 100 nM Cr(VI)	-24.23	-26.29 to -22.18	****	<0.0001
10 nM Cr(VI) vs. Ampicilline	30.83	28.78 to 32.89	****	<0.0001
10 nM Cr(VI) vs. Tetracicline	30.9	28.85 to 32.95	****	<0.0001
100 nM Cr(VI) vs. Ampicilline	55.07	53.01 to 57.12	****	<0.0001
100 nM Cr(VI) vs. Tetracicline	55.13	53.08 to 57.19	****	<0.0001
Ampicilline vs. Tetracicline	0.0667	-1.987 to 2.121	ns	>0.9999
***Klebsiella pneumoniae* ATCC 700503**				
Unexposed vs. 1 nM Cr(VI)	-1.133	-3.187 to 0.9208	ns	0.5861
Unexposed vs. 10 nM Cr(VI)	-3.867	-5.921 to -1.813	****	<0.0001
Unexposed vs. 100 nM Cr(VI)	-14.2	-16.25 to -12.15	****	<0.0001
Unexposed vs. Ampicilline	25.63	23.58 to 27.69	****	<0.0001
Unexposed vs. Tetracicline	25.4	23.35 to 27.45	****	<0.0001
1 nM Cr(VI) vs. 10 nM Cr(VI)	-2.733	-4.787 to -0.6792	**	0.003
1 nM Cr(VI) vs. 100 nM Cr(VI)	-13.07	-15.12 to -11.01	****	<0.0001

1 nM Cr(VI) vs. Ampicilline	26.77	24.71 to 28.82	****	<0.0001
1 nM Cr(VI) vs. Tetracicline	26.53	24.48 to 28.59	****	<0.0001
10 nM Cr(VI) vs. 100 nM Cr(VI)	-10.33	-12.39 to -8.279	****	<0.0001
10 nM Cr(VI) vs. Ampicilline	29.5	27.45 to 31.55	****	<0.0001
10 nM Cr(VI) vs. Tetracicline	29.27	27.21 to 31.32	****	<0.0001
100 nM Cr(VI) vs. Ampicilline	39.83	37.78 to 41.89	****	<0.0001
100 nM Cr(VI) vs. Tetracicline	39.6	37.55 to 41.65	****	<0.0001
Ampicilline vs. Tetracicline	-0.233	-2.287 to 1.821	ns	0.9994
***Staphylococcus aureus* ATCC 5538P**				
Unexposed vs. 1 nM Cr(VI)	-1.733	-3.787 to 0.3208	ns	0.1453
Unexposed vs. 10 nM Cr(VI)	-3.633	-5.687 to -1.579	****	<0.0001
Unexposed vs. 100 nM Cr(VI)	-18.53	-20.59 to -16.48	****	<0.0001
Unexposed vs. Ampicilline	22.43	20.38 to 24.49	****	<0.0001
Unexposed vs. Tetracicline	22.3	20.25 to 24.35	****	<0.0001
1 nM Cr(VI) vs. 10 nM Cr(VI)	-1.9	-3.954 to 0.1541	ns	0.0855
1 nM Cr(VI) vs. 100 nM Cr(VI)	-16.8	-18.85 to -14.75	****	<0.0001
1 nM Cr(VI) vs. Ampicilline	24.17	22.11 to 26.22	****	<0.0001
1 nM Cr(VI) vs. Tetracicline	24.03	21.98 to 26.09	****	<0.0001
10 nM Cr(VI) vs. 100 nM Cr(VI)	-14.9	-16.95 to -12.85	****	<0.0001
10 nM Cr(VI) vs. Ampicilline	26.07	24.01 to 28.12	****	<0.0001
10 nM Cr(VI) vs. Tetracicline	25.93	23.88 to 27.99	****	<0.0001
100 nM Cr(VI) vs. Ampicilline	40.97	38.91 to 43.02	****	<0.0001
100 nM Cr(VI) vs. Tetracicline	40.83	38.78 to 42.89	****	<0.0001
Ampicilline vs. Tetracicline	-0.133	-2.187 to 1.921	ns	>0.9999
***Enterococcus faecalis* ATCC 29212**				
Unexposed vs. 1 nM Cr(VI)	-0.233	-2.287 to 1.821	ns	0.9994
Unexposed vs. 10 nM Cr(VI)	-6.167	-8.221 to -4.113	****	<0.0001
Unexposed vs. 100 nM Cr(VI)	-12.33	-14.39 to -10.28	****	<0.0001
Unexposed vs. Ampicilline	23.33	21.28 to 25.39	****	<0.0001
Unexposed vs. Tetracicline	24.3	22.25 to 26.35	****	<0.0001
1 nM Cr(VI) vs. 10 nM Cr(VI)	-5.933	-7.987 to -3.879	****	<0.0001
1 nM Cr(VI) vs. 100 nM Cr(VI)	-12.1	-14.15 to -10.05	****	<0.0001
1 nM Cr(VI) vs. Ampicilline	23.57	21.51 to 25.62	****	<0.0001
1 nM Cr(VI) vs. Tetracicline	24.53	22.48 to 26.59	****	<0.0001
10 nM Cr(VI) vs. 100 nM Cr(VI)	-6.167	-8.221 to -4.113	****	<0.0001
10 nM Cr(VI) vs. Ampicilline	29.5	27.45 to 31.55	****	<0.0001
10 nM Cr(VI) vs. Tetracicline	30.47	28.41 to 32.52	****	<0.0001
100 nM Cr(VI) vs. Ampicilline	35.67	33.61 to 37.72	****	<0.0001
100 nM Cr(VI) vs. Tetracicline	36.63	34.58 to 38.69	****	<0.0001
Ampicilline vs. Tetracicline	0.9667	-1.087 to 3.021	ns	0.7355
